# Mr. Brande's Manual of Pharmacy

**Published:** 1825-07-01

**Authors:** 


*A Manual of Pharmacy.* By WILLIAM THOMAS BRANDE, F.R.S. &c. &c. Octavo, pp. 556. London, 1825.

To whom is the name of Brande unknown? To none who are either conversant with science or literature! Mr. Brande seems to have thought differently, and, as if he were one of the most obscure, has attached to his name no less than ten honorary appendages, being a regular enumeration of the offices which he fills, and of the societies which claim him for a member.

We conceive this to be in bad taste, and still worse judgment. It is a general and increasing practice with the members of the medical profession, to append to their names every little imaginary distinction which they may enjoy. We have long wished for an opportunity of expressing our sentiments on it, and of exposing its absurdity, and we thank Mr. Brande for the occasion his name affords us. The subject is less trifling than some of our readers may suppose, since it involves the judgment and respectability of numerous individuals.

The medical authors of this, or of any other age, may be conveniently divided into three classes. *First,* those of almost universal celebrity; *2dly,* men, perhaps equally gifted, but from age, situation, or other circumstances, less known; *3dly,* others altogether unknown beyond their circle, who write, not because they have a name, but to gain one. With respect to the first, to which Mr. Brande himself decidedly belongs, it is almost superfluous to state that they are physicians or surgeons to the King or Royal Family, attached to large hospitals, or are professors or public lecturers, for the world knows these facts well: but when they go farther still, and detail every petty circumstance connected with them, they render their titles ridiculous by such minuteness. What is it but the officiousness of men who are fearful they are unknown, forgotten, or unappreciated? What should we think of that man's sense who inscribed on a title-page "*William Shakspeare, Esq. F.A.S. of Stratford-on-Avon;*" or "*John Milton, A.B.;*" or "*Alexander Pope, A.M. and formerly President of the Literary Society of Twickenham?*" Can human folly extend farther than to obscure names which will for ever live, by adding to them common distinctions, indifferently bestowed on the talented and the worthless, the learned and illiterate? Are not the cases parallel? Who need be told that Brande is a professor, and member of various learned societies? or that Sir Astley Cooper is an hospital-surgeon, and Sir Henry Halford a physician to the King? If, however, justly celebrated men will append honorary distinctions to their names, let these be worthy of them; we only complain of those which are unworthy, because conferring no honour and easy of attainment. We are much pleased with the naked simplicity of a great name, and think it degraded by adventitious aids;—but this is a matter of opinion only. Neither Celsus nor Scipio Africanus requires an adjunct; they stand, unsupported, in isolated grandeur, mocking petty epithets.

In regard to the second and third classes, undoubtedly it is absolutely incumbent on them to place something after their names to inform the world who they are, and what have been their advantages; but when they descend to trifles they egregiously commit themselves. What then are the distinctions (the word is used conventionally) which medical authors of every rank may proclaim on their title-pages without a violation of taste and of judgment? Doctorates of medicine, memberships of surgical and medical colleges, official appointments to large hospitals or similar institutions to the blood-royal, professorships, public lecturers, fellowships of the Royal Society, Surgeoncies, or higher situations in the public service. Farther, or lower, no medical man can go, without raising a smile at his weakness. Who cannot but lament that a respectable man, deserving well of his profession, should gravely communicate to the public, that he was once a "President of the Medical Society of Edinburgh," or "a physician's clerk!" that he is now an "F.L.S." or "F.A.S." and "M.R.I.A." "Member of the Medico-chirurgical Society," or of the "United Physicians," or of several provincial societies, such as Hull, Manchester, or Newcastle! Happy individual to be so distinguished! Who cannot but smile, when an ingenuous youth confesses in his title-page, or preface, that he is a very young man, imperfectly educated,* an hospital mate, assistant-surgeon, or something still lower or humbler? What a recommendation to his readers! Did it never strike this very candid writer, that silence might have perpetuated a higher opinion of him; that had he withheld these too amiable weaknesses, he might have passed for a greater man? Do we recommend duplicity to authors? By no means! The public is capricious, easily swayed by name or rank, and no man ought to injure himself, and prejudice his readers against his book, by puerile acknowledgments.† It is the book, not the man, which is to be judged; let that book stand or fall by its own merits, but let it not suffer from the culpable candour of its author. Candour, excellent as it is, ceases to be a virtue, when it tramples upon common prudence and good sense. Do we treat with disrespect these humble situations? or the various literary societies? Certainly not! We think highly of them, we warmly recommend them; but we affirm they do no honour to medical men, and ought not to be officiously obtruded on the public eye. They are matters of course, and nothing more:—every reader gives a medical man credit for belonging to some of them,

* A fact! The author is now venerable and well known.

† The names of writers are concealed from delicacy; they are all before us.—*Rev.*

and demands no written proof. We are exceedingly anxious, that authors should appear before the world, in strict accordance with correct taste and sound judgment, seeking to exalt, and not to depress themselves;—thus imitating Julius Cæsar, who, when he fell basely murdered in the senate, carefully wrapped his robe around him, that he might fall, and die gracefully. Let it not be said, as far as our profession is concerned, in the words of the great moralist Johnson, that "*little things make little men proud.*" Every medical man cannot be great; Nature is too much of a niggard in her favours, and Fortune too sportive in her behests; but every one may imitate greatness, conduct himself with propriety and dignity, and confine himself within the precincts of taste and judgment. The world, however capricious at first, is generally ultimately just; never yet did any author attain an undeserved elevation without, at last, finding his level; and never did genius or learning fail to reach its goal, when malevolence and injustice were forgotten. Ponder on the fate of Darwin;—once he blazed like a meteor; where is he now? Gone to the "vault of all the Capulets;" no longer quoted; remembered only to "point a moral, or adorn a tale." Turn we to the lot of Sydenham. Nature was his guide, observation his polar star; opposition dogged his steps: theories clashed against his truths; yet he triumphed, and his name is eternal.

Mr. Brande, in his introduction, has explained his intentions so well, that we shall extract a part of it, and supply the remainder with a few comments.

"In a Manual of Chemistry, originally published in the year 1819, I have endeavoured to present the reader with a compendium of the principal facts of that science, arranged in the order in which they are discussed and illustrated in the Lectures delivered in the Laboratory of the Royal Institution. The object of the present work is to furnish the student with a corresponding outline of the Course of Pharmacy annually given at Apothecaries' Hall.

"Under the term Pharmacy I include all that relates to the Medical and Chemical History of the different Articles of the Materia Medica; to the mode of prescribing them; to their effects; to their composition: these therefore are the subjects to which I have almost exclusively confined myself in the following pages; and, it is hoped that they will be found so treated as to facilitate the progress of the medical student in a branch of his profession generally much neglected, and often imperfectly taught in the schools.

"Consistently with the plan of my Lectures, I have adopted the list of the Materia Medica, and of the Preparations and Compounds, sanctioned by the Royal College of Physicians of London, and published in their Pharmacopœia, as the basis of this work, and have endeavoured to divest my remarks upon them of all details, except such as I conceive likely to prove practically useful."—*Introduction, p.* x.

Mr. Brande concludes his introduction by three tables of weights and measures; and informs his readers, that medicines are compounded by Troy, and bought and sold according to Avoirdupois weight. He farther states that, "temperatures are, in all instances, measured by Fahrenheit's thermometric scale, in which the *boiling point* of water is 212°; the *freezing point*, 32°; by the term *gentle heat*, a temperature between 90° and 100° is implied; and the *specific gravities* of bodies are assumed as taken at 55°." We are much pleased with this minute information; it shews the author's good sense; a *manual* ought to descend to the most trifling matters. Systematic writers, and teachers of youth, err greatly in this particular, inasmuch as they presume far too much on the previous acquirements of their readers and auditors. They, who so commit themselves, are in the situation of Pope, who spoiled some of his finest epitaphs, from the omission of the names of the deceased. What good author would leave to accident, chance, or time, to supply a deficiency, which might be guarded against by the attention of a single moment?

The work of Mr. Brande is dedicated, with great propriety, to the Society of Apothecaries of London, and it consists of two parts. The first part "comprises the articles of the Materia Medica, the Latin names of which are arranged in alphabetical order." Part second "comprises the pharmaceutical preparations and compounds as directed by the London Pharmacopœia." Of a publication so various and complicated, it is obvious that a full and regular analysis would be impracticable, and, if practicable, misplaced; all we can do, is to select fair and appropriate specimens, to enable our readers to judge for themselves, and to follow them, with such corrections, observations, or additions, as may seem proper and suitable. It is the first part which now engages our attention, and this contains only 187 pages.

The two succeeding extracts are offered as specimens, and as specimens simply, of the mode of execution, and it is premised that the author never treats his subjects slightly or briefly, but usually with great ability and at considerable length. Indeed some of his articles are so long, yet not improperly so, that we cannot spare space for their citation.

"ALOES SPICATÆ EXTRACTUM—*The Extract of the Spiked Aloe, commonly called Socotrine Aloes.*—There can be little doubt, that the *aloe spicata* affords a very fine variety of the aloes of commerce, of which that imported from the Cape of Good Hope may be taken as a sample; but it is equally clear that the aloes met with in trade is of very different qualities, and although all the better kinds bear the name of Socotrine aloes, that they were never near the island whence it is professed they come.

"The fact is, that the greater part of the aloes we meet with is imported from Bombay, and not unfrequently remelted in this country, with a view of improving its tint and odour, and sometimes of deteriorating its quality by the addition of common rosin, a fraud easily detected by the insolubility of such adulterated aloes in boiling water: it is probably the inspissated juice or extract of several varieties of aloe; at all events, different samples differ remarkably in appearance and character.

"The finest aloes, that, for instance, of the island of Socotorah, and some of that prepared at the Cape, has a brilliant reddish-brown colour, and is very translucent at the edges of the fragmented pieces: its fracture is smooth and conchoidal, its odour aromatic and rather agreeable, its powder deep gold colour, its taste intensely bitter and nauseous. But such is rarely found at the druggists'; there it is more opaque, of a dull brown, often passing into black, of an odour generally decidedly disagreeable, and of a taste more than ordinarily nauseous. It is, however, scarcely necessary to enter at length into a description of these varieties of aloes, since their medical virtues do not seem to be essentially different, and accordingly we may select for pharmaceutical use that which has the least unpleasant odour, and which, at the same time, seems to have suffered least from heat in its manufacture, and which remains translucent.

"Aloes appears to be a mixture of gum, extractive, and resin, but whether its activity resides in one or all of these components has not been ascertained; nor, indeed, has much light been thrown upon its composition by those who have particularly examined it: it is nearly entirely soluble in boiling water, but, as the solution cools, some resin and altered extractive are thrown down; the alkalies and their carbonates form with it permanent solutions, and proof-spirit dissolves and retains it with only a slight precipitation of resin.

"The medical qualities of aloes are such as give it a place of its own in the Materia Medica. It is a warm stimulating purgative; its action is chiefly upon the large intestines, of which it singularly promotes the evacuation, probably by increasing the muscular or peristaltic action rather than by augmenting their secretions, for it rarely produces liquid motions. It generally sits well upon the stomach, and its bitterness promotes appetite and digestion; when in the small intestines it creates little alarm, and is seldom perceived till the sigmoid flexure of the colon feels its influence, the peristaltic movements of which are often perceptibly increased to the sensations of the patient, and then the rectum is quietly emptied.

"Much has been said of the mischief done by aloes in irritating the rectum, and no doubt it is liable to create excitement there, but this is only where it is frequently used, and in cases of habitual costiveness all other purgatives are open to the same objection. Sedentary, studious, and idle persons, and more especially females in the higher classes of society, resort to purgatives to obtain that regularity of intestinal evacuation which bodily exertion and due exercise only will insure; and aloes, in consequence of its moderate, but at the same time certain operation, is among the usual remedies thus erroneously employed; whence a portion of the ill fame which it has acquired as especially productive of piles and uterine and rectal irritation.

"In all cold indolent habits, where costiveness is attended by general sluggishness of the circulating system, with loss of appetite, irritability of temper, with disinclination both to mental and bodily exertion, and other symptoms of the milder hypochondriasis; where there is dyspepsia in females, blended with the disorders which arise from irregularity and inertness in the uterine system, aloes, in one or other of the forms I shall presently mention, is a valuable and safe remedy; and it is by far the most certain and secure substance for the relief of that temporary, but often obstinate and injurious costiveness, which usually follows the exhibition of opium.

"But there are cases in which aloetics are hurtful, and, to use a medical phrase, in which they are decidedly contra-indicated. Such, for instance, are plethoric and irritable habits subject to hæmorrhoidal affections, or to excessive uterine evacuations.

"The dose of aloes may vary from two to ten or fifteen grains; about five grains will usually evacuate the bowels in one or two bulky motions, but it is seldom that we give aloes alone, and under some of the aloetic formulæ of the Pharmacopœia, we shall again take occasion to speak of its most common combinations.

"The following pills are useful for obviating costiveness in dyspeptic habits, but they should not be kept too long in a dry place, as they are apt to become hard, and so insoluble as to pass through the bowels, —an inconvenience which may to a great extent be remedied by the addition to the mass of about a fourth part of sugar or of soap.

"R. Pulveris Aloës,
——— Mastiche,
——— Rhæi, aa ℨss.
Aquæ, q. s. ut fiant massa in pilulas xx. dividenda, quarum sumantur duæ vel tres ante prandium.

"The time for taking these pills is immediately before dinner; they then blend with the food, prevent flatulency, and are usually found to be operative the following morning after breakfast.

"ALOES VULGARIS EXTRACTUM—*Extract of the common Aloe; Barbadoes Aloes.*—This kind of aloes is prepared in Barbadoes, and exported thence in large gourds, which contain upwards of half a hundred weight each.

"It is generally deeper-coloured and more opaque than the former, its consistence is tougher, its fracture less shining, and its odour strong and peculiarly disagreeable; the colour of its powder is dirty yellow, and it is said to be more active than Socotrine aloes, and hence, though its price usually exceeds that of the other varieties, it is much preferred in the preparation of horse medicines, a channel by which enormous quantities of aloes are consumed." 17.

"AMYGDALA AMARA } DULCIS } *Varieties of the Amygdalus communis*—*Bitter and Sweet Almonds.*—Both these kernels agree in containing albumen, mucilage, and a considerable proportion of a bland, insipid, and inert oil (amounting to between 45 and 50 *per cent*), easily obtained by expression; but to these ingredients there is superadded in the bitter almond a principle which gives it its peculiar flavour, and which may be obtained by distillation with water; it then appears a volatile oil, generally heavier than water, having the concentrated odour of the bitter almond, and partaking of some of the chemical properties of the hydrocyanic acid. It is this ingredient which renders bitter almonds intensely poisonous to some animals, and not unfrequently they produce deleterious effects upon the human system.

"The distilled oil is virulently active, and the symptoms attendant on poisoning by it are in some respects marked and peculiar. They have been thus enumerated by Dr. Granville*: 'Stupor and numbness, with oppression and a sense of weight at the summit of the head; yawning and an irresistible disposition to sleep; vertigo, and dizziness of sight. All or any of these preliminary symptoms, according to the quantity of the poison taken, are generally observed by the practitioner, if sent for in time. The pulse is found to be rather strong at first, but flags soon after, and becomes either frequent, wiry, and small, or slow and vibrating. A paralytic state of the extremities is next remarked, the pupil remains unalterably dilated, the sensibility of the organs of sense is greatly diminished. Every animal function seems impaired, except respiration, which is very rarely indeed accelerated or difficult. Vomiting and hiccup shortly precede the aggravation of every nervous symptom, when life ebbs fast and becomes at last extinct.'

"From Mr. Brodie's experiments to ascertain the mode in which death is immediately produced by this and analogous poisons, it would appear that they operate upon the nervous system; that through the medium of the nerves the influence of the poison is conveyed to the brain, the functions of which are more or less impaired; that the organs of respiration are thus secondarily affected, but that the action of the heart continues for a long time unimpaired, circulating venous blood: hence, if respiration be artificially performed, so as to aërate the blood, it sometimes happens that the animal permanently recovers. Upon these subjects the reader is referred to Mr. Brodie's papers in the Philosophical Transactions, and to Orfila's Traité des Poisons.

"* Treatise on Prussic Acid, 1820, p. 89."

"Essential oil of bitter almonds is largely prepared for the use of perfumers, confectioners, and cooks, who generally use what is called the essence of almonds, or a solution of ʒi. of the oil in ʒvj. of alcohol; this is also the most convenient form for its pharmaceutical employment. One hundred weight of the bitter almond cake remaining in the press after the separation of the fixed oil, is put into the still with about 400 gallons of water, this large proportion being necessary to prevent the formation of a mucilaginous magma, from which the volatile oil will not pass off, and which often, if brought to boil, rises up into the head and worm of the still. The produce of oil is liable to much variation, 1 cwt. of cake yielding from 2 ounces to 2¼ by weight. It often deposits a considerable portion of white crystallised matter, which is apparently a peculiar vegetable compound.* The oil appears to be composed of hydrocyanic acid in union with volatile oil. By digesting red oxide of mercury in it, Mr. Hennell obtained cyanuret of mercury, from which pure hydrocyanic acid was as usual procured by distilling it with muriatic acid.

"The cases in which it has been proposed to administer this oil, are those in which the diluted hydrocyanic acid has been recommended, and of which a full account will be found in Dr. Granville's 'Historical and Practical Treatise.' Affections of the lungs, preceding or connected with phthisis; coughs of a spasmodic character, and especially whooping cough; an irritable state of the nervous system; asthmatic complaints, and some cases of local irritation; are the principal diseases in which it has been found useful, and it may be administered with camphor and other antispasmodics.

"There is so much difference in the strength of the diluted hydrocyanic acid usually sold for medical use, that it is difficult to state any precise dose in which it may be administered. That made at Apothecaries' Hall has a specific gravity of 995, and contains 3.2 *per cent* of real acid: upon this subject, Dr. Ure's observations in the Quarterly Journal† may be consulted with advantage. From 1 to 10 or 12 minims of such diluted acid may be regarded as the limits of its dose, but it is right to commence its use with caution, and gradually to increase the quantity given until an effect is observed.

"The action of this acid, as well as of the volatile oil of almonds, depends much upon its state of concentration: of the latter, from 1 to 8 drops or minims in an ounce or an ounce and a half of any proper vehicle may be generally safely given. If sense of faintness, attended by nausea, chills, giddiness, or dimness of sight, should ensue, the dose must be diminished. Among others, the following formulæ for the exhibition of oil of bitter almonds may be used—

"℞ Olei Essent. Amydgd. Amar. ♏viij.

Mucilaginis Arabici ʒij.

Misturæ Camphoræ f℥vj.

M. fiat mistura, de qua sumantur cochlear duo ampla bis vel ter die.

"* Quarterly Journal, vol. xv. p. 376." "† Vol. xiii. p. 313"

"The hydrocyanic acid, or the essential oil of almonds, may be added to the mixture prescribed at page 4, in cases of cough and catarrhal irritation of the larynx.

"Sweet almonds, triturated into a paste with sugar and a little water or mucilage, as directed for the *confectio amygdalarum* of the Pharmacopœia, and afterwards properly diluted and strained, as in the *mistura amygdalarum*, furnish a pleasant emulsion which may be used merely as a diluent drink, or as a vehicle for active remedies. In warm weather it becomes sour in the course of 24 hours, and should therefore always be used fresh.

"The expressed oil of almonds in the formula above referred to furnishes an elegant emulsion, and its tastelessness recommends it in preference to olive oil. With the alkalies it forms a soapy mixture which may be substituted for emulsion, or which sometimes, with an increased quantity of alkali, is used in renal and urinary irritation, especially that arising from uric sand.

"℞ Olei Amygdalarum f℥ss.

Aquæ Rosæ f℥ij.

Liquoris Potassæ fʒj. Misceantur agitatione, et adde

Syrupi Simplicis f℥ss.

Aquæ Distillatæ f℥v.

M. fiat mistura, de qua sumantur f℥ij. pro dosi.

"In cases of catarrh, with hoarseness, fʒiss. of liquor ammoniæ, is sometimes substituted in the above mixture for the solution of potassa." 24.

The preceding extracts will furnish a good idea of Mr. Brande's work, and as in the last, he adverts to the hydrocyanic acid, we shall offer a few remarks on it. We have before us, most opportunely, a letter from a much valued friend, and excellent physician, in the West Riding of Yorkshire, in which, without our having ever adverted to such a subject, he says—"In a case of pulmonary consumption, the hydrocyanic acid was carried to its utmost limit. I was excessively disappointed with it; for, the only effect I could ever discover, as produced by its exhibition, was, an extraordinary irregularity of the pulse, with frequent intermissions. I wish to put you on your guard against the seductive reports of Dr. Elliotson, and other advocates."—Our experience of this medicine has been very confined; we never knew it to do either good or harm; it appeared to act like an anodyne; and this was the opinion of a patient himself, who imagined he had taken opium.

Mr. Brande's account of copaiba is meagre, imperfect, and incorrect. It shall be given without abridgement, and to it certain observations will be appended.

"COPAIBA—*The liquid Resin of the Copaifera officinalis,* vulgarly called *Balsam of Copivi.*—This liquid resin, obtained by wounding the bark of the tree, is imported from the Brazils in small casks. It has the consistency of oil, but is more viscid and glutinous; a pale yellow colour; a peculiar and somewhat aromatic odour, disagreeable only to those who have taken it as a medicine; and a pungent nauseous taste. Its specific gravity is 950. It communicates flavour to water shaken with it, and is perfectly soluble in about eight parts of alcohol, and in ether in any proportion. When rubbed upon paper and dried, it leaves an apparently greasy stain, but this differs from that of oil, by admitting of being written over with common ink. If adulterated with oil the stain would of course be truly greasy, but I have rarely met with any samples which I have seen reason to believe either spurious or sophisticated; though the appearance of the article as it occurs in trade differs considerably, yet this is probably owing to the circumstances under which it is collected.

"The term *balsam* being now generally restricted to compounds of resin and benzoic acid, is not applicable to this substance; nor is it strictly a liquid resin, but a compound of volatile oil and resinous matter. When distilled, the former, which is highly odorous and pungent, and upon which the virtues of copaiba depend, passes over, and there remains an insipid resin in the retort, which has sometimes been employed in medicine under the name of *inspissated copivi balsam.*

"Copaiba has long been celebrated in the cure of gleets, fluor albus, and other similar discharges unattended by active inflammation; and more lately it has been found effective in the relief of hæmorrhoidal affections. Its principal operation is diuretic, and in large doses it proves gently aperient. Where there is inflammatory action going on any where in the urinary canals, this medicine should be avoided; yet I have seen it of service in allaying the irritation and diminishing the secretion of red or uric sand.

"It may be given in doses of from fifteen to forty drops twice or thrice a day, either upon water, or triturated into an emulsion by the aid of yolk of egg or gum arabic. One drachm has been administered three times a day in piles, and in this dose it generally purges. It is apt to nauseate, and I think more so when given in emulsion than when simply swallowed with plain water, but the effect may, in some degree, be prevented by the addition of some aromatic water to the emulsion of copaiba, as in the following:—

"R Mucilaginis Acaciæ ʒiss.

Copaibæ ʒss. tere simul et adde gradatim

Aquæ Menthæ viridis ℥j.

Tincturæ Capsici ♏v.

M. fiat haustus, bis vel ter quotidie sumendus." 17.

Mr. Brande altogether overlooks the notorious fact of copaiba having been employed by certain eminent authors in chronic affections of the larynx and trachea, and for which it promises to be an excellent remedy, since it exercises a remarkable effect on all mucous membranes, checking their morbid secretion, and restoring them to perfect sanity. Even some ancient writers recommend copaiba for pulmonary complaints, and if we are not mistaken, Morgagni, with a mixture of sulphur. We think Mr. Brande is somewhat inconsistent when he restricts its exhibition during an inflammatory action of the urethra, and acknowledges he has "seen it of service in allaying the irritation, and diminishing the secretion of red or uric sand."—We are of opinion, that this medicine, a drachm every night and morning, may be safely administered during the inflammatory stage of gonorrhœa, and with palpable success. Under such treatment, an incipient gonorrhœa has ceased in two or three days; and obstinate gleets have yielded to it in fifty hours; yet the dose should be continued for a considerable time afterwards. We never knew any thing but benefit result from this practice, and an eminent surgeon, who, at our suggestion, has employed it for eight years, confirms the truth of this statement. It was Dr. Dawson* of Sunderland, who first adopted this mode of treatment on an extensive scale; he communicated it to his friend Dr. Armstrong,† and other gentlemen, who, after a fair trial, became its warm supporters. The following is the formula advised.

℞. Copaibæ, syrupi zingiberis āā ℥i.

Mucilaginis acaciæ.

Aquæ puræ āā ℥iij.

Fiat mistura.

Sumatur uncia una omni nocte et manè.

The mixture often purges; this is an advantage, but may be easily corrected by opium, if desired. Often have we seen, and so has a friend of more extensive practice, two doses of this preparation actually remove a copious urethral discharge of several months duration. We consider the formula of Mr. Brande to be exceedingly objectionable from the improper introduction of the *tinctura capsici.* He seems to have thought that a mixture of contraries would

"Make the gruel thick and slab:"

*Shakspeare, Macbeth, Act* IV. *Scene* I.

We differ again from our respected author, where he tells us, the copaiba is apt to nauseate more in an "emulsion than when simply swallowed with plain water." The truth is, an emulsion, or mixture is the most pleasant form, and the swallowing of it upon water the most disgusting. Great names cannot weigh

* Author of the Nosological Practice of Physic; reviewed in our Journal for April, 1825. See the work, page 170.

† Consult Armstrong's Practical Illustrations of Scarlatina, &c. London, 1818.

against personal experience, nor fanciful opinions against damning truths. Away with arguments;—let us come to facts. Did Mr. Brande ever take any copaiba? He alone can answer this question; the probability is he never did. Now we have, at one time of our life, swallowed ounces of copaiba; and, therefore, his mere *ipse dixit* cannot sink the personal experience of years, backed by the practice and sentiments of many friends, together with our own, in hospitals and private life. Much of the efficacy of copaiba depends on the mode of administration. This medicine, in conjunction with water, or mixed with spirits of nitre, has been taken for a long series of years up to the present day for gleets or leucorrhœa, and with little advantage, if the reports of patients are to be credited. No mean part of the benefit is derived from the happy admixture of copaiba with water and mucilage, or yolk of egg. Gentlemen have applied to us, have said they had tried copaiba without any success; yet, unknown to themselves, they have obtained a rapid cure from the same article under another and better form. No man was more partial to copaiba than Dr. Daniel Turner* of London, whose name the *ceratum calaminæ* sometimes bears; he only used it for gleets, and in the form of a paste, made with sugar; yet his cures were surprising, and there can be no deception, since individual cases are narrated. For hæmorrhoids copaiba is doubtless a valuable remedy, and it may be added, in all affections resulting from an irritative or inflammatory state of mucous membranes. But for the nauseousness of copaiba, and the disagreeable eructations which it usually causes, we should pronounce it to be the very best purgative for habitual constipation; for, in a few hours, without either uneasiness or griping, it brings away immense quantities of fæces, whose consistence is moderately fluid. We really wish our readers would give a fair trial to the copaiba mixture in gonorrhœa, gleets, leucorrhœa, and chronic affections of the larynx, trachea, bronchia, and rectum, and obligingly communicate the results to the editor.

Mr. Brande has written very well on opium; yet the subject is so interesting as to induce us to supply certain omissions or deficiencies observable in the article.

We have frequently expressed our opinions of this drug as a medical agent, and, in this instance, we shall, therefore, confine our observations to somewhat novel matter.

Opium exercises a wonderful effect on the human body, and every circumstance connected with it deserves especial notice.

* Vide his Treatise on the Venereal Disease, &c.

Our experience of it has been great indeed, and it shall be briefly communicated. It is the only medicine which most certainly controls or subdues pain; it does more than this, it imparts to the mind a delightful tranquillity, and, hence, it becomes doubly a favourite. With idiosyncrasies of constitution we meddle not, since they are as various as the winds, but, in the generality of constitutions, an adequate dose overcomes pain and excites happiness within an hour. Unfortunately, like all other stimulants, for a moderate quantity is a stimulus, and an immense one a sedative, it loses its effect by daily repetition, and fifty drops of the tincture may, within twelve months, be advanced to eight or nine drachms, with scarcely a similar result. The exhilirating effect of opium is very unlike that of wine or spirits; for the head is unaffected, the faculties bright and clear, the body active and vigorous; and, although the person seems to pass the night without sleep, yet still he is pleasant and comfortable, arises happy and refreshed, and remains so until the period of his next dose:—then, and not till then, he feels that there is something wanting also, a sinking of the stomach, and considerable languor. It is no less singular than true, that if a man accustomed to a large nocturnal dose of opium, swallows a somewhat minor quantity, he, notwithstanding he sleeps well, leaves his bed weak, languid, and without an appetite, and which will continue for several hours; while, on the contrary, if he take a full dose, appear to sleep scarcely any, and roll from side to side incessantly, yet he is in the morning, cheerful, hungry, and invigorated. Are then the days of opium-takers all halcyon ones? Alas! they are the reverse, and beset with evils, which shall be explained. The dose requires a gradual increase; this is a serious matter, for it soon, in a year or two, becomes so large as to need a division of it into two or three doses, which mostly occasions a slight nausea of an hour's duration, inasmuch as this medicine decidedly impairs the powers of the stomach. Opium, in long continued quantities, usually produces obstinate constipation, demanding almost constant purgatives. After dinner, from disturbed sleep by night, the patient is excessively drowsy; scarcely able to read; and naturally sleeps in his chair for hours, if undisturbed; during which sleep, he sometimes, but not always, imagines, and really seems to feel that he is convulsed or paralytic; and, when he awakes, he is languid, unhappy, and miserable. He remains in this state for an hour or two; next takes his opium; and, before two hours have elapsed, all is sunshine!—he is happy and vigorous, fit for deeds of arms, or great literary undertakings. Nor is this a fallacy; men actually act, speak, and write better under the influence of opium; hence its liberal use among the Turks, as well as celebrated public characters. Bad as the practice is, and we admit it to be very bad, yet still it is not half so deadly as immoderate drinking. Unfortunately, when an individual has been long accustomed to the delights of opium, he seldom or ever abandons it, for the want of it is the acmé of human misery. His spirits are gone, his activity fled, his appetite reduced; to him the world is a blank, its allurements empty names; he is really sunk into feebleness; he crawls from place to place like a victim to typhous fever; the muscles of his loins and limbs are aching and powerless; his only wish is to lie down and stir no more; yet his usual dose will, in an hour, restore him to happiness, health, and strength; and this no other human agent can accomplish, since, to an opium-taker, wine or spirits have a contrary effect, and soon nauseate. Doubtless, these unpleasant consequences will gradually disappear, provided the sufferer persevere in his abstinence, but they are so dreadful, and of such long continuance, that he seldom gains a triumph over his inclinations, and when, to such sufferings, pain is superadded, he is too often almost compelled to return to his favourite remedy. We are well acquainted with a distinguished instance, in which the subject displayed a degree of resolution worthy of a Roman name. For nearly four years this gentleman, in other respects healthy, suffered a very frequent pain of his stomach, which rendered life nearly a burthen. Every remedy having been tried in vain, and his days and nights usually consumed in excruciating pain, but without vomiting, except on one occasion, and no bad health; he, in despair, took 60 drops of tincture of opium by night, increased the number, found the relief he sought, and being forced to continue the opium, for the pain invariably came on nightly, he incautiously ran up the dose to nine drachms in eleven months; and this he regularly persevered in for thirteen months longer, vacillating between seven drachms for his *minimum*, and nine for his *maximum*. During these two years the pain was never considerable, and as he suffered all the evils which have been enumerated, he determined to conquer a practice so pernicious, although it assuredly conferred on him ease and comfort. It may be interesting to write the scale by which he effected his design, premising that he invariably took the medicine in the evening, and never at any other period.

*T. Opii. Saturday*, ʒvij. *(continued, more or less, for nearly* 2 *years.)—Sunday*, ʒv.—*Monday*, ʒv.—*Tuesday*, ʒiv.—*Wednesday*, ʒiv.—*Thursday*, ʒiij.—*Friday*, ʒijss.—*Saturday*, ʒij.

and, after this, the task was easy, yet he continued this dose for a few weeks, to guard against a recurrence of pain in his stomach. Thus it will be evident that, in seven days, this gentleman omitted the enormous quantity of 23½ drachms of tincture of opium. It may be urged he proceeded too rapidly; he did so, but he was the occasional subject of pain, and it was absolutely necessary to catch the golden periods, since a severe attack might have rendered every resolution fruitless. In lieu of the *extra quantum* of opium, he, during slight pain and sinking of the stomach in the evening, which were accompanied with universal lassitude, drank gradually a pint of warm ale, and this greatly revived him, besides being an indispensable auxiliary. This gentleman, ever studious and temperate, succeeded at last;—his sufferings, they have been already described, were most distressing and heart-rending; but they slowly yielded to time. His appetite for breakfast was reduced from two biscuits to half a biscuit; and, to use his own language, "I have succeeded; the advantage I have derived is problematical; upon the same terms, another such victory would be dearly purchased, for the good of it is not very evident!" This is not the language of a valetudinary, but of a man in apparent health, whose mind has been enlightened by education and science, and is, therefore, entitled to respect and attention. We have before remarked,* that opium might be more extensively used for the alleviation of pain resulting from chronic diseases; indeed, it is admissible for every kind of pain, except, perhaps, that of inflammation, and, even here, it is not without able advocates;† and it should be administered in suitable doses. Two important cautions it is our duty to inculcate, and which should never be disregarded. *First;* Opium ought not to be exhibited simply to raise the spirits, a practice most ruinous; but should be confined to the removal of severe pain, or such other purposes as judgment sanctions. *Secondly;* Never, on any occasion whatever, should the dose, for one day, exceed three drachms of the tincture, or a corresponding quantity of the drug itself. Within these limits, the administration of opium is safe, agreeable, and efficacious:—beyond them there is a sea of trouble and danger.‡

* Review of the Confessions of an Opium-eater.

† See Dr. Armstrong's paper, Transactions of the Associated Apothecaries and Surgeon-Apothecaries of England and Wales, vol i. London, 1823.

‡ To prevent misconception, which often prevails where prejudice operates, we distinctly declare that this gentleman sustained no injury from opium, but obtained a decided and permanent benefit. When he commenced its use, there was every probability that life would be exhausted by

Mr. Brande offers little that is interesting respecting the well known drug—alum. The analysis of Dr. Scudamore's publication* on the blood, and on the powers of a saturated solution of alum, as a styptic remedy in hæmorrhage, and as an internal medicine in hæmorrhagic affections, fell to the lot of a more able and learned colleague than ourselves, yet, as Dr. Scudamore invited his professional brethren to communicate their experience of this remedy, we shall dwell on it briefly. Alum has always been a great favourite with us, and we have seen it prescribed, and directed its exhibition, during a period of nearly thirty years. As a styptic for bleeding vessels, it has long been deservedly notorious; and it is rather surprising that this author should have imagined, as he seems to imagine, that there was much novelty or merit in his saturated solution, for alum and water have ever been employed for such purposes. The old surgeons preferred to mix the alum with the albumen of an egg, and, in this state, applied it with success to bleeding wounds, and alum is the only effective agent in the old "*pulvis stypticus*" of the shops. For years we have recommended injections of solutions of alum for hæmorrhages from the uterus. A solution of alum remarkably corrugates the mucous membrane of the vagina; and, in one case, where this canal was of large size, from repeated parturitions, it, under the application of this solution, became so contracted as scarcely to admit a finger. We have seen great benefit derived from the internal employment of alum in hæmoptysis and menorrhagia. With us the dose is fifteen grains thrice a day; the alum having been finely powdered, and either exhibited in powders, boluses, or pills. In this way we lately cured, and that in a few days, a case of menorrhagia, which had resisted every remedy prescribed by a judicious accoucheur.

The first part of this work shall be concluded with a short yet good article on the leaves of senna.

"SENNÆ FOLIA—*The Leaves of the Cassia Senna.*—The European market is supplied with senna leaves from Alexandria, whither they are brought from Upper Egypt; and after having been mixed and adulterated with leaves of the *cynanchum oleafolium*, or *argel*, and occasionally also with the leaves of bladder senna, box, and some others, they are packed in bales for exportation.

---

anxiety, pain, and sleeplessness; yet, under the administration of it, the pain became insignificant, other gastric symptoms were removed, health and vigour followed; and, although he certainly severely suffered from its discontinuance, yet still he was comparatively well of the complaint, which demanded the exhibition of opium.—*Rev.*

" It is difficult to describe the characters which should guide us in the selection and purchase of senna; among them we may enumerate a bright fresh colour, and an agreeable smell somewhat resembling that of green tea. It should not be too largely mixed with stalks, seed pods, and other extraneous matter, nor very much broken, nor very dusty.

" Senna has, when chewed, a nauseous flavour quite peculiar to itself; with boiling water, (which, according to Mr. Thomson, dissolves about a third of the weight of the leaves employed,) it affords a brown infusion, very nauseous both in smell and taste, and liable to decomposition. Proof spirit dissolves a larger portion of senna, and forms a brown active tincture. Alcohol and ether yield green solutions.

" Senna has been chemically examined by Bouillon la Grange.* The effects of various re-agents on infusion of senna have been described by Mr. Battley.† According to Lassaigne and Feneulle, the activity of senna, as a purge, depends upon the presence of a peculiar vegetable principle, which they have termed *cathartine*, and which may be procured as follows:—To an aqueous decoction of senna leaves add subacetate of lead as long as it occasions a precipitate, which is to be washed, diffused through water, and subjected to a current of sulphuretted hydrogen gas. Separate by filtration, and reject the precipitated hydrosulphuret of lead. Evaporate the clear liquor to dryness, digest the residue in alcohol, and again evaporate to dryness. This alcoholic residue contains acetate of potassa, which may be decomposed by the addition of sulphuric acid, and the sulphate of potassa separated by filtration. Then add acetate of lead to precipitate the sulphuric acid, and pass sulphuretted hydrogen through the liquid; filter, evaporate, and cathartine remains. It is deliquescent, and uncrystallisable, of a reddish colour, a bitter taste, insoluble in ether, but soluble in water and alcohol.‡

" The above process is so circuitous, and the vegetable principle subjected to such a variety of agents, that no dependence can be placed upon the supposed existence of its product as a distinct pre-existing principle of senna.

" Senna is rarely to be depended upon alone, for it generally occasions griping, spasmodic, and flatulent pains of the bowels, without duly evacuating them; but it is a truly valuable auxiliary to other purgatives, and in many such mixtures clears the bowels with speed and certainty. The infusion is the best form for its exhibition; and ginger, as in the *infusum sennæ compositum*, is a good addition. It should be recently prepared, for if kept exposed to air it undergoes a change, and is said to be more apt to gripe. To these formulæ the tincture of senna may be added, and the following is one of the many modes of prescribing them:—

" * See Annales de Chemie, vol. xxxvi. p. 3."

" † London Medical Repository, vol. xv. p. 169."

" ‡ Annales de Chemie et Physique, tom. xvi. p. 16."

"R Infus. Sennæ f℥iv.

Magnes. Sulphatis ℥j.

Aquæ Menthæ sativ. f℥ij.

Tinctur. Sennæ f℥ss.

M. sumat cochlear. iv. mane primo et repet. post horas tres, si opus sit.

"The addition of syrups to such mixtures only renders them more nauseous, without assisting their efficacy: half an ounce of manna is, however, occasionally added to the above. Soluble tartar and infusion of senna form a good active purge, and pimento water covers much of the disagreeable flavour.

"R Potassæ Tartratis ʒj.

Infus. Sennæ compos.

Aquæ Pimento aa fʒvj.

Tinct. Jalapæ fʒj.

M. ft. haust. laxans." 160.

We now arrive at the second part of the Manual of Pharmacy, or that which treats of preparations and compounds, amounting to 342 pages. This part alike correct and instructive, contains, as has been stated, the London Pharmarcopœia, the Latin and English being placed in divided and parallel columns. To each preparation or compound, Mr. Brande attaches valuable observations, chiefly chemical and medical, and which will well repay an attentive perusal. Here our author is quite at home; handles, explains, and comments on every thing which passes in review before him, with all the facility and talent of a master; and his admirers will no more expect error from such a quarter, than the too credulous Romans did from the oracular responses of their favourite temples. "Tunc divinam speciem et vim respondi ex nomine *Basilidis* interpretatus est."*

Agreeably to our usual practice, we shall select a favourable example of the manner and execution of the second part, which will convey a more lively idea than grave description.

"PREPARATION OF SILVER.

| "*Argenti Nitras.* | "*Nitrate of Silver.* |
|---|---|
| R Argenti unciam,<br>Acidi nitrici fluidunciam,<br>Aquæ destillatæ fluiduncias duas; | Take of Silver, an ounce,<br>Nitric Acid, a fluidounce,<br>Distilled Water, two fluidounces; |
| Acidum nitricum Aquâ misce, et in his Argentum balneo arenæ liqua. | Mix the nitric Acid with the Water, and dissolve the Silver in the |

* Taciti Historiæ, Lib. iv. Cap. lxxxii.

| | |
|---|---|
| Dein calorem paulatim auge, ut siccetur Argenti Nitras. Hanc in crucibulo, lento igne, liquefac, donec, expulsâ Aquâ, cessaverit ebullitio; tum statim effunde in formas idoneas. | mixture in a sand-bath. Then increase the heat gradually that the Nitrate of Silver may be dried. Melt this in a crucible over a slow fire, until, the Water having been expelled, the ebullition ceases; then pour it immediately into proper moulds. |

"In this process the silver is oxidized at the expense of one portion of the acid, nitrous gas is therefore evolved, and the resulting oxide of silver (composed of 110 silver + 8 oxygen = 118) combining with the other portion of the acid yields, on evaporation, colourless prismatic crystals of nitrate of silver, which are anhydrous, and consist of—

| | | |
|---|---|---|
| "1 proportional of Nitric Acid ........ | 54. ....... | 31.4. |
| 1 ———— Oxide of Silver...... | 118. ...... | 68.6. |
| | 172. | 100. |

"For internal use this salt ought to be kept in crystals, but the above formula directs the immediate evaporation of the solution to dryness, and the fusion of the dried salt, which is then cast into moulds for surgical use; and it is in this state only that it is usually found in apothecaries' shops.

"It is better in all cases to crystallise the nitrate of silver, and carefully to fuse the crystals in a silver crucible over a lamp: the mould in which the sticks are cast should be warmed to prevent the too rapid cooling of the fused salt, which then becomes extremely brittle; and care should be taken to employ no more heat than is requisite for its liquefaction, since it easily suffers decomposition and becomes black from the deposition of oxide of silver. The silver employed should be free from copper, and the acid should be pure, in which case the resulting solution is clear and colourless; a little black powder frequently separates, which is gold. This salt of silver is eminently susceptible of the agency of light, which blackens and decomposes it.

"The principal use of nitrate of silver is as a caustic; it kills the parts to which it is applied, and being much less soluble than pure potassa, and not deliquescent, it is easier of application and less apt to spread. Distilled water at 60° dissolves about its own weight of this salt; the solution should be transparent and colourless; if of a bluish tinge, and becoming deep blue on the addition of ammonia, it contains copper. It may thus be used in any state of dilution, and is a very valuable application in many cases of ulcerating sores, in the proportion of from one to five grains to the ounce of distilled water. The part may be touched twice or thrice a day with a camel-hair pencil dipped in this solution, which should be of such strength as to occasion smarting. In fistulous sores it is sometimes used as an injection, and it has been recommended as a mouth-wash in scorbutic affections of the gums, and aphthæ of the fauces.

"As an internal remedy nitrate of silver has gained much and apparently deserved celebrity in the treatment of epilepsy. In this disease it has been administered in doses beginning with an eighth of a grain and carried up to four or six grains three or four times a day; it is usually taken made into pills with bread-crumb, and the best dose appears to be half a grain thrice a day, gradually increased to a grain and a half or two grains. Under this treatment the fits often decrease at first in violence and then in frequency until the patient recovers, and where the bowels are moderately acted upon the efficacy of the remedy appears most certain.* There is a very disagreeable effect which frequently follows this use of nitrate of silver, which is the discoloration of the *rete mucosum*, so that the whole surface of the body, and especially those parts which are most exposed to light, acquire a leaden-grey or livid-colour which is permanent.† Various means have been resorted to with a view of preventing this effect, or of removing it when it has taken place, but hitherto without success. It is curious that excessive acidity at the stomach is a frequent concomitant of epilepsy, and that Dr. Prout's experiments‡ have shewn that the free acid of the stomach is the muriatic, an acid the base of which would instantly decompose the nitrate of silver.

"Nitrate of silver has occasionally been resorted to in other diseases attended by morbid nervous excitement and debility; in certain convulsive affections, in chorea, and in *angina pectoris*.

"When taken in over-doses, it operates in the same manner as the other corrosive poisons; perhaps the best antidote consists in copious draughts of salt and water, by which chloride of silver would be formed, a compound, in all probability, nearly, if not quite inert. Orfila's results, however, in reference to this counter-poison, were not very satisfactory.§" 265.

Before we make another extract, we shall, in the way of variety, as painters mingle light and shade, move forwards, and select a short passage, for a reason which will soon be apparent.

"*Buckthorn is only fit for a veterinary pharmacopœia. The syrup is never prescribed, and, consequently, if found in an apothecary's shop, is always in an unfit state for use.*" 478.

This is at once unqualified, positive, and dictatorial; and, considering the high authority which becomingly attaches to the name of Brande, will be believed by numerous individuals. Now nothing can be more incorrect, or more unworthy of a man who is eminently qualified to instruct youth. We beg to tell Mr. Brande that he is mistaken, and would recommend him to be more cautious. His respectability renders his errors dangerous, and the teacher of others should himself be correct. Syrup of Buckthorn is prescribed and kept in the shops of apothecaries;

---

"* Med. Chir. Trans. vol. ix. p. 234."

"† Albers and Roget, Med. Chir. Trans. vol. vii. p. 284."

"‡ Phil. Trans. 1823. § Traité des Poisons, tom. i. part ii. p. 46"

an ounce acts as an excellent purgative; and, in smaller doses, it is occasionally directed for children. It is only a month ago, while attending a child of six years old, with a very respectable surgeon, that we prescribed it; and we were assured by this gentleman that he frequently administered it as a laxative to children with considerable success. Surely this will shew that Mr. Brande has written unguardedly. He says, it "is always in an unfit state for use." How does he prove this? To his mere assertion we oppose respectable authority; the syrup purges and the patient recovers; what more decisive or conclusive can be adduced?

A second interesting extract shall be offered, as more likely to elucidate the nature of the work, and the information it embraces, than simple analysis.

"Acetate of lead forms prismatic crystals, soluble in about four parts of water, at 60°, and of a singularly sweet and somewhat astringent taste. It generally occurs in commerce in masses composed of irregular assemblages of acicular crystals, and is seldom perfectly soluble in water. The smallest portion of sulphuric or carbonic acid in water produces a precipitate when acetate of lead is added. A current of carbonic acid, passed through a solution of this salt, throws down one half of the oxide of lead in the state of carbonate, and an uncrystallisable *binacetate* of lead remains in solution. When finely powdered protoxide of lead is boiled in a solution of the acetate, a portion equal to that originally existing in the salt is taken up, and a *subacetate* of lead is formed, composed of one proportional of acid and two proportionals of oxide: in the above formula, therefore, rather less carbonate of lead than is required to saturate the acid is directed, in order to insure the production of the neutral acetate.

"Externally, acetate of lead has been very long employed as a sedative and astringent application; it has also acquired considerable celebrity as an internal remedy, requiring, however, considerable caution in its exhibition. Its principal use is in *urgent* cases of internal hæmorrhage, as of the lungs, stomach, or uterus; and, in consequence of the violently spasmodic action of the bowels, which it is apt to induce, it requires generally to be given with opium, and often with some mild aperient. Paralytic affections occasionally follow its administration, where due precaution in regard to the state of the bowels is not taken.

"In hæmoptysis where the quantity of blood coughed up is considerable, or where the usual remedies, especially nitre and dilute sulphuric acid, fail, acetate of lead may be given as in the following formula, care being taken to avoid, in the medicines and drinks, those acids which decompose it, and especially the sulphuric, by which, as appears from Orfila's experiments,* it is rendered nearly, if not quite inert:

"* Traité des Poisons, tom. i. 2me partie, p. 281"

hence it is that sulphate of soda is an effective antidote where any of the soluble salts of lead have been swallowed.

"℞ Plumbi Acetatis, gr. iv.

Pulveris Opii, gr. iij.

Confectionis Rosæ Caninæ, q. s.

Misce et divide in pilulas sex æquales, quarum sumatur una ter in die.

"A proper dose of digitalis may be combined with the above pills; or it may be given with acetate of lead as follows:—

"℞ Plumbi Acetatis, gr. j. Solve in

Aquæ Rosæ f℥j. et adde

Oxymellis Simplicis ʒfj.

Tinctur. Opii ♏v.

——— Digitalis ♏x.

Fiat haustus quartis vel sextis horis sumendus.

"In hæmatemesis the same remedies may be had recourse to; also in menorrhagia; but in all these cases the necessity of active measures for subduing febrile symptoms, where the inflammatory diathesis prevails, must not be lost sight of; nor should the patient be, in any case, suffered to continue the use of the acetate of lead for a length of time.

"When solutions of acetate of lead are used externally, the salt should be dissolved in distilled water, and all substances tending to decompose it should be carefully avoided. The addition of a little acetic acid to these lotions prevents the deposition in them of carbonate of lead; and where they are used as collyria, such deposition is sometimes mischievous: they are employed in ophthalmia, and generally as astringents and sedatives in all cases of superficial inflammation. When, as is often the case, a little carbonate of lead is contained in the acetate, the solution should generally be directed to be filtered, especially if it is to be applied to the eyes. For collyria, the proportion of acetate of lead may be about ten grains, and for lotions about thirty grains, to eight ounces of rose or elder flower water, and two or three drachms of distilled vinegar may be added." 309.

Mr. Brande, writing of the *tinct. ferri muriatis,* proceeds thus:—

"As a tonic, from five to thirty drops may be taken twice a day in a wine-glass of water. In dyspepsia, small doses are generally more serviceable than larger ones, and it may be given in any bitter infusion, those being generally selected which are not blackened by it.

"℞ Tincturæ Ferri Muriatis ♏vj.
Infusi Quassiæ,
Aquæ Cinnam, aa f℥vj.
Tincturæ Calumbæ, f℥j.
M. fiat haustus mane et meridie sumendus.

"The dose may gradually be augmented, if necessary, to thirty drops, but it is apt, in larger doses, to produce head-ach, to harden the pulse, and occasion slight spasmodic pains of the stomach and bowels.

If it constipates, a drachm of sulphate of magnesia may be added to each draught. If it produces diarrhœa, the bowels should be cleared with a little rhubarb and magnesia, and it may then be resumed generally without ill effect.

"As a tonic, after diseases of debility or depletion of the system, it requires, as well as the other preparations of iron, considerable circumspection in its use; if it induces no local inflammatory action or headach, and neither hardens the pulse, nor materially quickens it, it often proves eminently active in restoring tone to the constitution.

"With aloetics and antispasmodics it is not an ineffective emmenagogue, though not, upon the whole, so well calculated to fulfil this particular indication as some of the other chalybeates. It may be given as follows:—

"R Tinct. Ferri Muriatis,
Tinct. Aloes compos.
Tinctur. Valerianæ, aa f℥ss.
M. sumatur cochleare unum minimum ex infusi anthemidis cyatho, bis vel ter quotidie.

"Like other chalybeates, it sometimes expels worms from the intestines. It is used as a styptic in internal hæmorrhages. In scrofulous affections it is a most valuable tonic, especially when it can be given in large doses. Mr. Thomson recommends the dose, in these cases, to be gradually increased up to 120 drops twice a day.

"In retention of urine, depending upon spasmodic stricture of the urethra, this solution of iron has been represented as having something like a specific action. Five or six drops have been recommended in such cases, by Mr. Cline, every ten minutes, until nausea is produced.

"As an external application, the '*tinctura ferri muriatis*' has been applied to cancerous and other very ill conditioned sores, but not with any marked success." 282.

There is much of error and misconception in the above extract. Spasmodic stricture is an incorrect term, and ought to be abolished. What is a stricture? A more or less alteration of structure in a canal. Now, spasm of the urethra occasions no structural change, consequently, the word stricture is misused, in defiance of all true logic. Retention of urine sometimes arises from general or partial spasm of the urethra, and it should be denominated a spasmodic affection, and not a stricture. If Mr. Brande had introduced as many catheters as we have, and if, during a strong spasmodic action, he had taken hold of the instrument while the urine was discharging, and felt the violent and grasping manner in which it is embraced by the urethra, he would scarcely have employed the appellation of stricture; and this circumstance is, in our opinion, an almost demonstrative proof of the muscularity of that canal, notwithstanding the assertions of men who stand high in their own estimation as well as in that of the public. In truth, there is a great want of precision in the language of medical men in general, who appear to use words more for sound than as symbols of definite ideas. Vaccination is a fertile source of error in this respect. We read of vesicles and pustules; and, strange to relate, lymph springs from the pustules, and pus out of the vesicles. While errors and inconsistencies so glaring insult the understanding, surely it is our bounden duty to expose and correct them; and it is our invariable custom, and always shall be, never to cite examples without well knowing the authors who have so committed themselves, yet their names are, and probably ever will be by us, scrupulously concealed. Mr. Brande says five or six, but he is mistaken, it is twenty, or even thirty drops of the *tinct ferri. mur.* which Mr. Cline recommends, and he does not mention nausea, but until a sensible effect is produced; and this phrase may, perhaps, have misled our author into a belief that nausea was the effect contemplated. It is scarcely to be expected that a man, agonized by retention* of urine, could be nauseated by only five or six drops of the tincture every ten minutes. In the language of Hotspur, he would not have "*time to be sick!*" This medicine is only admissible in spasm of the urethra; in inflammation, it would be highly dangerous; in stricture, absurd; yet it is too often prescribed on such occasions. It is not long ago since a young physician ordered the *tinct. ferri muriat.* for a man whose urethra was altogether impervious from strictures, and who was eventually cured by the knife. This is practice without principles. Sound principles are the

* Even this obvious term and its companion, suppression of urine, are, in utter defiance of correctness, frequently used by authors as synonimous and convertible designations. *Retention* implies that the urine is *retained* within the bladder: whilst *suppression* indicates that the secretion of urine is *suppressed*;—two most opposite states of disease; being the ischuria vesicalis and renalis of systematic writers. Mr. Hey has pointed out these distinctions in his surgery, but no originality belongs to him, for they were particularly dwelt upon by Mr. Cline, in his lectures, long before he published them. It is impossible to mention the venerable name of Cline, without a hasty and feeble attempt to sketch his character. We knew him well, yet twenty one years have elapsed since we witnessed his successful labours. As an operator, he was neat, cool, and determined: as a lecturer, we never met with his equal or superior. He was always mild, grave, sensible, and dignified;—he seldom praised, and never censured: he was never eccentric or sarcastic: to him, vanity, arrogance, or self-sufficiency were strangers. His language was chaste and correct; his judgment sound; his caution proverbial; his manners and appearance gentlemanly. He was constitutionally reserved and solemn; every one felt that he could not presume to break in upon the privacy of Mr. Cline, but while he did so, he also felt, that it was the privacy of an amiable, scientific, and honourable man, and, as such, entitled to his respect and esteem.—*Rev.*

ground-work of the medical art; and slow and cautious observation of what passes before the eyes is the superstructure. Without these, it is mere empiricism; and the worst of empiricism, because it abuses mistaken confidence, and sports with human life. In fact, if practitioners, after having gained correct principles of their profession, would only use their eyes and ears, carry common sense to the bed-side, exercise a cool and steady judgment respecting what they see and hear, and act accordingly, they would succeed infinitely better;—but, unluckily for themselves, and unfortunately for others, they are so confined by authorities, so shackled by dogmas, so enchained by opinions, that they surrender their own personal experience and are misled, when they might have been enlightened. It may seem paradoxical to assert, but we contend for its truth, that learned men are generally the worst practitioners and practical writers, and this obviously arises from the very causes which we have stated and condemned. The term "*learned*" is employed in the most scholastic sense; and the word "*unlearned*" with a liberal interpretation, implying a person acquainted with English, Latin, French, and a little Greek, but still not deserving to rank with distinguished scholars, who are alone learned, and of whom the amiable John Mason Good is a bright example. Learned men, whether practising or writing, are, from their intimate acquaintance with authors of every description, so bewildered by astounding names and conflicting testimonies, that they seldom act or think independently. It is rare for such men to produce truly original works; for as, after all, there is little novelty under the sun, they are fearful to think for themselves, and timid to promulgate opinions, which, under different modifications, they suspect may be found in ancient writers, and thus it is they most commonly descend to mere compilation. Unlearned men, on the contrary, are infinitely less read, and know less of medical lore, hence, they act more boldly and think more independently; and, when they write, proceed fearlessly, not contemplating imputed plagiarisms from works of antiquity. John Hunter himself is a strong example; learning would have impeded his acute mind, and retarded his prolific pen;—what he saw, thought, or did, he proclaimed—totally ignorant that much of his lucubrations and sentiments was discoverable in books to him unknown or inaccessible. Learning enlarges the mind, dissipates its vanities, and softens its asperities; but it damps its ardour, while it curtails its original, and augments its accumulative powers. This compliment, however, we can unfeignedly pay to learned authors, that if their writings be less original, they are most certainly far more modest and engaging. The records of this, and of every other age, would convincingly prove the truth of the foregoing statements; yet we decline to bring forward names, especially of the illustrious living, which might give offence where none is intended.

We can only afford space for another extract, and with which our review shall be concluded, after we have indulged in a few observations. Our limits have been exceeded by a natural desire to impart useful information, which we prize more highly than fastidious criticism.

"As an internal remedy, sulphate of zinc, is chiefly used as a tonic and emetic; applied externally, it is a powerful and often useful astringent. It is given in very small doses, a quarter of a grain, for instance, twice or thrice a day, in dyspepsia (see page 146); in epilepsy one or two grains have been given every four or six hours; and in all diseases of debility connected with inflammatory action, it has been preferred to other mineral tonics, as being less apt to excite thirst, arterial action, and other febrile symptoms; but I doubt whether there are good grounds for this preference.

"In dyspeptic affections, sulphate of zinc and other similar and powerful remedies generally require to be given in very small doses, and to be long persevered in, if we wish to derive the utmost benefit from their powers. Dr. W. Philip says of this remedy—'in the opinion of many, the sulphate of zinc, given in very small doses, holds a distinguished place among the astringents suited to indigestion, and it is sometimes successful where other tonics fail. It may be given at later periods than iron, but it requires caution, and if its good effects do not soon appear, should be laid aside. It is one of those powerful agents which must always be employed with some degree of suspicion.'*

"Sulphate of zinc is one of the numerous remedies resorted to in the cure of epilepsy, where it is usually given with vegetable bitters, and with the more powerful antispasmodics.

"℞ Zinci Sulphatis gr. j.
Extracti Anthemidis gr. x.
Misce fiant pilulæ duæ quater die sumendæ.

"The following has also been much extolled in epilepsy, by those who confide in the virtues of musk:—

"℞ Zinci Sulphatis gr. x.
Moschi ʒj.
Camphoræ ℈j.
M. et divide in pilulas viginti, quarum sumantur duæ bis vel ter in die.

"In diseases attended by considerable irritability as well as debility, sulphate of zinc certainly appears preferable to sulphate of iron. In the

"* On Indigestion, 4th edit. p. 206."

advanced period of whooping cough, from an eighth to a quarter of a grain may be given twice or three times a day, conjoined with small doses of infusion of bark, or of cascarilla; or it may, if requisite, be united in pills with extract of hemlock or of henbane. 'In spasmodic coughs,' Dr. Paris says,* 'it is administered with the best effects, especially when combined with camphor or myrrh.

" R Zinci Sulphatis gr. x.

Myrrhæ in pulverem tritæ ʒjss.

Confect. Rosæ q. s. ut fiant pilulæ viginti, è quibus sumantur binæ bis quotidie.

"'In affections of the chest attended with inordinate secretion, I have witnessed much benefit from its exhibition, particularly when presented in the form of lozenge.'

"In the cure of intermittent fevers, sulphate of zinc is an admirable tonic, either with or without Peruvian bark; and in all those obstinate cases where the use of arsenic has been suggested, this salt of zinc should have a previous trial.

" R Zinci Sulphatis gr. ij.
Aquæ Cinnamomi,
——— destillatæ, aa f℥ijss.
Tincturæ Calumbæ f℥j.
M. fiat mistura cujus capiat æger cochlearia tria ampla tertiâ vel quartâ quâque horâ.

"It has been said that sulphate of zinc, which occasions a precipitation in infusion of Peruvian bark, is therefore incompatible with it; but the combination remains very effective: thus—

" R Zinci Sulphatis gr. ss.

Decocti Cinchonæ fʒxv.

Tincturæ Gentianæ compos. ʒj.

M. fiat haustus ter quaterve die sumendus.

"Nor does the following appear to be an unchemical combination—

" R Zinci Sulphatis gr. ¼.

Quiniæ Sulphatis gr. ij.

Infusi Rosæ compos. fʒx.

Tincturæ Aurantii,

Syrupi Aurantii, aa fʒj.

M. fiat haustus quartâ quâque horâ sumendus.

"In ulcerated sore throat a gargle of sulphate of zinc is often of the greatest local service: the following is among other forms that may be used:—

" R Zinci Sulphatis ℈j.

Aquæ Rosæ f℥vij.

Oxymellis Simpl. f℥j.

M. fiat gargarisma frequenter utendum.

"* Pharmacologia, vol. ii. p. 462."

"As an emetic, sulphate of zinc is generally very rapid and very certain in its action, and well suited to those cases in which it is desired to empty the stomach of any poisonous contents. The average dose for this purpose is twenty grains.

"℞ Zinci Sulphatis ℈j

Aq. Menthæ viridis f℥jss.

Spt. Lavandulæ compos. fℨj.

Fiat haustus emeticus.

"At the commencement of febrile diseases, and in other analogous cases where emetics are administered, tartarised antimony and ipecacuanha, which tend to produce diaphoresis, independent of the mere exertion of vomiting, are to be selected in preference to sulphate of zinc.

"In the form of a dilute solution in distilled water, sulphate of zinc is a good astringent application; as such it is used in ophthalmia, after the vascular congestion and excessive irritability have been removed, and when the inflammation tends to become chronic.

"℞ Zinci Sulphatis gr. x.
Aquæ Rosæ f℥viij.
M. fiat collyrium.

"A similar or somewhat stronger solution may also be employed with advantage in the latter stages of gonorrhœa." 318.

To render the work still more useful Mr. Brande has added a posological table, a second of pharmaceutical equivalents, and a very ample and convenient index.

It will have been obvious from our copious extracts, and the attention we have bestowed, that we entertain a favourable opinion of this work. We warmly recommend it to our readers. Such a Manual of Pharmacy, emanating from an eminent individual, ought to be in the hands of every medical student, in the surgeries or shops of every surgeon or apothecary, and the first men of the profession will not refuse a place to it in their libraries, if they are actuated by utility and judgment in the selection of their books.

